# Comparative Analysis of Volatile Organic Compounds in Freshwater-Cultured and Saline–Alkaline Selectively Bred Tilapia Using Electronic Nose, GC-IMS, and HS-SPME-GC-MS

**DOI:** 10.3390/foods14223946

**Published:** 2025-11-18

**Authors:** Zhi Wang, Yi Yang, Dongxue Zhang, Jiashu Li, Longsheng Zhang, Yan Zhao, Jinliang Zhao, Junling Zhang, Jikui Wu

**Affiliations:** 1 Shanghai Engineering Research Center of Aquatic-Product Processing & Preservation, Shanghai Ocean University, Shanghai 201306, China; wzhi2022@126.com (Z.W.); 13479396924@163.com (Y.Y.); 13399587098@163.com (D.Z.); ljs18003502814@163.com (J.L.); 2 Key Laboratory of Exploration and Utilization of Aquatic Genetic Resources, Ministry of Education, Shanghai Collaborative Innovation Center for Aquatic Animal Genetics and Breeding, Shanghai Ocean University, Shanghai 201306, China; zlsheng0208@163.com (L.Z.); y_zhao@shou.edu.cn (Y.Z.); jlzhao@shou.edu.cn (J.Z.); 3 Laboratory of Quality and Safety Risk Assessment for Aquatic Product on Storage and Preservation (Shanghai), Ministry of Agriculture, Shanghai Ocean University, Shanghai 201306, China

**Keywords:** tilapia, salt-alkali water, volatile organic compounds, E-nose, GC-IMS, GC-MS

## Abstract

Tilapia is a cornerstone species in global aquaculture, yet the impact of saline-alkaline adaptive breeding on its flavor-related volatile organic compounds (VOCs) remains unclear. Herein, we compared VOCs in freshwater-cultured tilapia (FW) and 7th-generation tilapia subjected to long-term selective breeding for saline-alkaline tolerance (SAW_G7_) using an electronic nose (E-nose), gas chromatography-ion mobility spectrometry (GC-IMS), and headspace solid-phase microextraction-gas chromatography-mass spectrometry (HS-SPME-GC-MS). The aim was to identify flavor differentiation and assess the effect of saline-alkaline acclimation. E-nose analysis revealed distinct odor profiles, with SAW_G7_ showing higher sensor responses for aldehydes, ketones, and alcohols. GC-IMS detected 32 VOCs, highlighting significant increases in alcohols, aldehydes, and heterocyclics in SAW_G7_. GC-MS identified 43 VOCs, with orthogonal partial least-squares discriminant analysis (OPLS-DA) confirming 18 discriminant compounds, including elevated ketones (2-undecanone), aldehydes ((E)-2-octenal), alcohols (2,7-Octadien-1-ol), and furans (2-ethyl-Furan) in SAW_G7_, linked to lipid oxidation under saline-alkaline stress. These findings demonstrate that long-term saline-alkaline breeding achieves a potentially more diverse VOC profile in tilapia by altering its volatile profiles. The study provides insights for optimizing aquaculture practices to improve product quality in marginal environments.

## 1. Introduction

Globally, salinization impacts approximately 1.125 billion hectares of land, with human activities contributing to roughly 76 million hectares [1]. In China, saline-alkaline soils cover about 99.13 million hectares, concentrated in the northeast, northwest, and coastal zones [1]. Utilizing inland saline-alkaline water for aquaculture presents a strategic approach to conserve freshwater resources and expand production within affected regions, despite the challenging environment for most aquatic species [2,3].

Certain species, such as *Litopenaeus vannamei* and *Carassius auratus gibelio,* have been successfully cultured in saline-alkaline waters. Nevertheless, the quality of aquaculture animals is closely related to their rearing environment. Saline-alkaline water significantly influences the nutritional composition and flavor profiles of these animals. For instance, long-term low-salinity culture of the mud crab (*Scylla paramamosain*) results in a notable increase in unsaturated fatty acids and the content of sweet amino acids in the crab’s muscle tissue. Similarly, saline-alkaline cultivation enhances the quality of Chinese mitten crab (*Eriocheir sinensis*), while inland low-salinity saline-alkaline environments significantly elevate the nutritional and umami profiles of red drum (*Sciaenops ocellatus*) muscle tissue [4,5,6].

Nile tilapia (*Oreochromis niloticus*), ranking second globally in aquaculture production, exhibits exceptional adaptive plasticity and disease resilience, underpinning its emergence as a genetically selectable candidate for saline-alkaline aquaculture systems [7,8]. In 2023, our research team achieved a breakthrough by selectively breeding the first Nile tilapia (*Oreochromis niloticus*) strain adapted to inland saline-alkaline waters in China. We previously demonstrated that saline-alkaline stress remodels the digestive and absorptive functions of this species, leading to quantifiable alterations in the protein and lipid profiles within muscle tissues [9]. However, the flavor profile and underlying quality formation mechanisms of tilapia acclimated in these specific conditions remain systematically unresolved. This constitutes a critical knowledge gap, as the unique ionic composition and pH of saline-alkaline water may fundamentally alter the fish’s metabolic homeostasis, lipid oxidation, and accumulation of flavor precursor compounds, thereby shaping its volatile organic compound profile and taste characteristics. Elucidating the flavor fingerprint of saline-alkaline bred tilapia is therefore not merely a question of sensory evaluation, but a central scientific problem for achieving a sustainable saline-alkaline fishery that moves beyond survival to producing high-quality, consumer-preferred products. Consequently, a systematic investigation into these flavor attributes is essential to guide precision feeding strategies, optimize aquaculture protocols, and ultimately enhance the commercial value of the final product.

The principal flavor components in aquatic products comprise volatile odorants and nonvolatile taste-active substances. Volatile odorants, critical determinants of aquatic product aromas, include aldehydes, alcohols, ketones, esters, nitrogen-containing compounds, sulfur-containing species, and heterocyclic aromatics [10,11]. These compounds originate from enzymatic, microbial, and abiotic pathways within aquatic matrices, directly determining the olfactory and gustatory sensory attributes [12,13]. Contemporary aquatic product volatile organic compound (VOC) analysis predominantly employs singular methodologies, such as electronic nose (E-nose), gas chromatography-mass spectrometry (GC–MS), or gas chromatography-ion mobility spectrometry (GC–IMS), each presenting distinct analytical trade-offs. While E-nose technology enables rapid flavor profiles through cross-reactive sensor arrays, its inability to resolve specific compounds limits mechanistic insights [14]. Conversely, GC–MS offers exceptional compound identification precision yet suffers from limited sensitivity toward trace-level volatiles [15]. GC–IMS provides operational simplicity, enhanced sensitivity for short-chain oxygenates, and visual data representation, albeit with semi-quantitative limitations [16]. Thus, the reliance on singular analytical approaches creates significant limitations in comprehensive flavor assessment, particularly in evaluating the flavor changes of tilapia cultured in freshwater versus saline-alkaline environments.

Herein, we conducted a comparative analysis of VOCs in freshwater-farmed tilapia (FW) and 7th-generation tilapia bred for long-term salinity-alkalinity tolerance (SAW_G7_) using E-nose, GC-IMS, and GC-MS [17]. This study aimed to identify key volatile compounds distinguishing FW and SAWG7 and assess the impact of saline-alkaline acclimation on flavor complexity. Our findings provide novel insights into the compositional basis of tilapia flavor, supporting the optimization of aquaculture practices to enhance product quality.

## 2. Materials and Methods

### 2.1. Samples and Reagents

The tilapia samples used in this study comprised freshwater-farmed tilapia (FW) and 7th-generation tilapia from long-term salinity-alkalinity-tolerant selective breeding (SAW_G7_). The SAW_G7_ tilapia strain was developed through seven consecutive generations of selective breeding with saline-alkaline water containing sodium chloride (171 mmol/kg) and sodium bicarbonate (23.8 mmol/kg) at the Zhongjie National Tilapia Breeding Farm (ZNTBF) in Hebei Province, China.

This experiment was conducted in July 2024 at the ZNTBF. The experimental samples consisted of standardized, healthy, and disease-free FW (body weight: 245.26 g ± 13.45 g) and SAW_G7_ (body weight: 250.48 g ± 11.87 g), which were used for subsequent experiments. The FW (*n* = 30) and SAW_G7_ (*n* = 30) were acclimated for one week in 400 L white PVC plastic barrels before the experiment began. Two groups were established, each with three replicates, and each replicate contained 10 fish. The fish were reared in 400 L white PVC plastic barrels (96 cm × 80 cm × 71.5 cm) filled with underground well water for a four-week feeding trial. Throughout the experiment, feed was provided at 2.5% of the fish’s body weight daily at 9:00 AM and 5:00 PM. All feed used was Aohua brand extruded feed for polyculture fish. The water temperature was maintained at 24 ± 0.5 °C, and sufficient dissolved oxygen was ensured.

All tilapia specimens (average weight: 500 ± 50 g) were sampled from ZNTBF (Staufen, Germany). The fish muscle was sectioned into 1 cm^3^ cubes, minced by a grinding machine (IKA A11 basic Analytical mill, Staufen, Germany), rapidly frozen in liquid nitrogen, and stored at −80 °C in a laboratory freezer to preserve its biochemical properties until subsequent analysis [18]. Additionally, 2,4,6-Trimethylpyridine (TMP, 99%, chromatographic reagent) was sourced from Sinopharm Chemical Reagent Co., Ltd. (Shanghai, China) and employed as the internal standard. All other analytical-grade reagents were purchased from Aladdin Biochemical Technology Co., Ltd. (Shanghai, China).

### 2.2. Electronic Nose (E-Nose) Analysis

The odor profiles of the tilapia samples were analyzed using a Fox 4000 electronic nose system equipped with an 18-channel sensor array (Alpha M.O.S., Toulouse, France). This methodology was adapted with minor modifications following the approach established by Nie et al. [19]. Each minced fish meat sample (2.0 g) was placed into 10 mL headspace vials. The heater parameters were as follows: incubation time, 25 min; incubation temperature, 85 °C. The headspace injector needle settings were cleaning time, 120 s; injector temperature, 50 °C; injection volume, 2400 μL; injection speed, 2000 μL/s. Acquisition settings comprised: 120 s acquisition time and 600 s delay time. The blending speed was adjusted to 500 rpm. Three replicates were recorded for each sample.

### 2.3. Volatile Organic Compounds (VOCs) Detection Based on Gas Chromatography-Ion Mobility Spectrometry (GC-IMS)

The GC-IMS system employed in this study was obtained from G.A.S. (Dortmund, Germany). The analysis utilized the MXT-WAX capillary column (RESTEK, Bellefonte, PA, USA), measuring 30 m × 0.53 mm, and high-purity N_2_ (Nitrogen, 99.99%) as carrier gas. The operational parameters for the GC-IMS were as follows: The column temperature was held constant at 60 °C. Initially, the flow rate was established at 2.0 mL/min for 2 min, which was then ramped up to 10 mL/min over the next 8 min and subsequently increased to 100 mL/min within 10 min. Each sample underwent a total run time of 30 min. The detection temperature in IMS was maintained at 85 °C, with the high-purity N_2_ serving as the drift gas. For the analysis, each minced fish meat sample weighing 2.0 g was incubated in 20 mL headspace vials at 60 °C for 20 min while being mixed at a speed of 500 rpm. Afterward, 500 μL from each vial was automatically injected at 85 °C using automatic injection mode. All analyses were conducted in triplicate to ensure reliability.

### 2.4. VOCs Determination Using Headspace Solid-Phase Microextraction Coupled with Gas Chromatography-Mass Spectrometry (HS-SPME-GC-MS)

An adapted version of the methodology described by Shi et al. was adopted [20]. Each meat sample (5.0 g) was blended with 5.0 mL of saturated NaCl solution to create a uniform slurry and then transferred to a 20 mL headspace vial. The sample was spiked with an internal standard, 2,4,6-trimethylpyridine (TMP), at a concentration of 10 μg/kg. The samples were analyzed using an Agilent 6890 system (Agilent Technologies, Santa Clara, CA, USA) equipped with mass spectrometry and an Agilent DB-WAX (Agilent Technologies, CA, USA) capillary column (60 m × 0.25 mm × 0.25 μm). Volatile compounds were extracted by headspace solid-phase microextraction (HS-SPME) using a 50/30 μm PDMS/DVB/CAR fiber assembly (Supelco, Bellefonte, PA, USA). The fiber was conditioned at 270 °C for 0.5 h prior to its initial use and re-conditioned before each analysis following the manufacturer’s protocol. Helium (He, 99.99%) served as the carrier gas. Helium (He, 99.99%) served as the carrier gas. Each sample was incubated in a vial at 60 °C for 10 min with agitation at 500 rpm to reach equilibrium. Subsequently, the conditioned fiber was exposed to the vial headspace and extracted for 40 min at 60 °C. The fiber was then rapidly inserted into the GC injection port and thermally desorbed at 250 °C for 3 min. The GC oven temperature program was as follows: initial temperature held at 40 °C for 10 min; increased to 180 °C at a rate of 5 °C/min; then raised to 230 °C at a rate of 20 °C/min and held for 8 min.

The volatile compounds detected through GC-MS were identified by comparing their mass spectra against the NIST database, with only those exhibiting a spectral match quality above 80% being retained for further analysis. Quantification was performed using the internal standard method with a multi-point calibration curve. Calibration standards were prepared at concentrations ranging from 1 to 500 μg/kg for all analytes. The concentration of the internal standard in each sample was 10 μg/kg.

### 2.5. Odor Activity Value (OAV) Analysis

Compounds present at high concentrations with low odor thresholds are potential candidates for characteristic aroma components. The Odor Activity Value (OAV), defined as the ratio of a compound’s concentration to its odor threshold, is a key metric for evaluating its actual impact. When OAV is less than 1 (OAV < 1), the compound’s contribution to the overall flavor is generally not significant. Conversely, when OAV is greater than 1 (OAV ≥ 1), the compound is considered aroma-active and has a direct impact on the overall flavor profile. The OAV is calculated as follows:

$$OAV=\frac{C}{T}$$

where:

OAV: Odor Activity Value
*C*: Compound Concentration
*T*: Odor Threshold

### 2.6. Data Analysis

For HS-SPME-GC-MS, VOCs were qualitatively analyzed using mass spectrometry (MS) in conjunction with the retention index (RI). Unknown compounds were identified by referencing the NIST 17 database. The RI values were calculated based on the retention times of C7-C40 n-alkanes. The quantitative analysis of VOCs was performed by the internal standard method.

IMS data were processed with specialized spectral interpretation tools comprising the LAV 2.0 software suite (G.A.S.), the Reporter visualization module, the Gallery Plot comparative analysis system, and GC-IMS spectral matching algorithms, enabling multidimensional data interpretation. Compound identification followed a dual-parameter validation protocol integrating chromatographic retention indices with ion mobility separation metrics, systematically cross-verified against the NIST reference database and proprietary IMS spectral repository maintained by G.A.S., according to established analytical chemistry standards.

Significant differences were assessed utilizing SPSS 16.0 software. Principal component analysis (PCA) and Orthogonal Partial Least-Squares Discriminant Analysis (OPLS-DA) were conducted using SIMCA 14.1. Furthermore, additional plots were created using Origin 2024 software.

All experiments were conducted in triplicate. Data from different stages were compared using one-way ANOVA, followed by Bonferroni’s post hoc test for multiple comparisons, with significance set at *p* < 0.05.

## 3. Results and Discussion

### 3.1. E-Nose Analysis of FW and SAW_G7_

**Figure 1:**
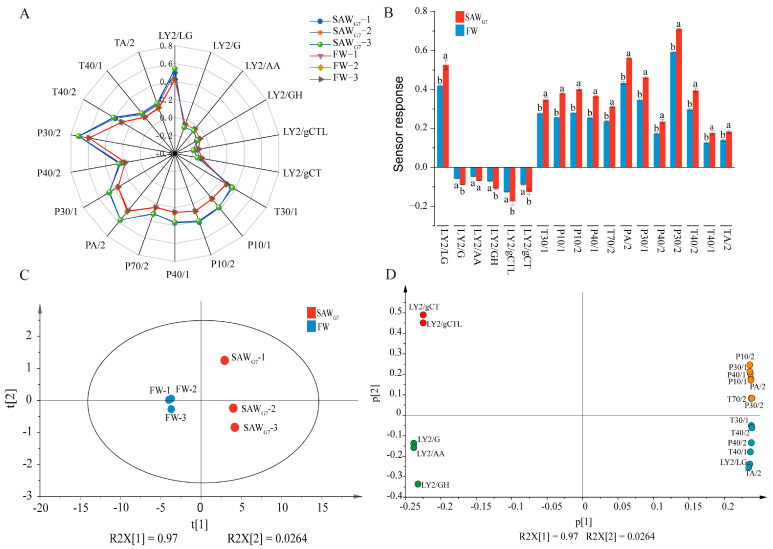
E-nose analysis. (**A**) Radar chart of the E-nose for SAW_G7_ and FW. (**B**) Difference analysis of sensor response between SAW_G7_ and FW. Bars labeled with the same lowercase letter are not significantly different, whereas those with different letters are significantly different (*p* < 0.05). Error bars represent standard deviation. (**C**) Principal component analysis (PCA) plots of SAW_G7_ and FW. (**D**) Load analysis of E-Nose sensor response values.

We utilized the E-nose to analyze the odor characteristics of FW and SAW_G7_. The sensor array comprises multiple sensing elements, each exhibiting distinct responsiveness to different volatile compounds (Table S1). Radar plot analysis demonstrated that each sensor exhibited distinct responses upon exposure to headspace volatiles from FW and SAW_G7_, while odor profiles of both samples demonstrated notable similarities (Figure 1A). As illustrated in Figure 1B, LY2-type sensors predominantly displayed negative values, except LY2/LG, which was sensitive to aldehydes. Positive responses were detected mainly by P- and T-type sensors. The P30/2 and T40/2 sensors recorded the highest positive values, which specifically captured ketones and polar alcoholic compounds, respectively [21,22]. Among the 18 sensors, SAW_G7_ exhibited consistently higher sensor response intensities than FW. All sensors except LY2/AA showed statistically significant differences (*p* < 0.05), suggesting the levels of aldehydes, ketones, polar alcoholic compounds, and other compounds in SAW_G7_ changed during long-term saline-alkaline adaptation.

Principal component analysis (PCA) was performed on the electronic nose (E-nose) sensor response values for FW and SAW_G7_. The PCA plot revealed different clustering patterns: FW samples occupied the left quadrant, whereas SAW_G7_ samples clustered in the right quadrant. The cumulative contribution rates of PC1 (97%) and PC2 (2.64%) accounted for 99.64% of the variance (Figure 1C). Loading analysis further discriminated the relative contributions of individual sensors to the differentiation of volatile components between FW and SAW_G7_, as visualized by sensor positions in Figure 1D. Seven sensors (P10/2, P30/1, P40/1, P10/1, PA/2, T70/2, P30/2) primarily influenced PC1, while two sensors (LY2/gCT, LY2/gCTL) were predominantly associated with PC2. These findings suggested that ketones, alcohols, alkanes, and sulfur compounds critically differentiate the volatile profiles of FW and SAW_G7_. The results confirmed distinct flavor profiles between the two samples, with the E-nose effectively distinguishing them. To assess variations in volatile compound profiles of FW and SAW_G7_, gas chromatography-ion mobility spectrometry (GC-IMS) and gas chromatography-mass spectrometry (GC-MS) were employed to identify and quantify flavor-related constituents systematically.

### 3.2. VOCs in FW and SAW_G7_ Identified by GC-IMS

**Figure 2:**
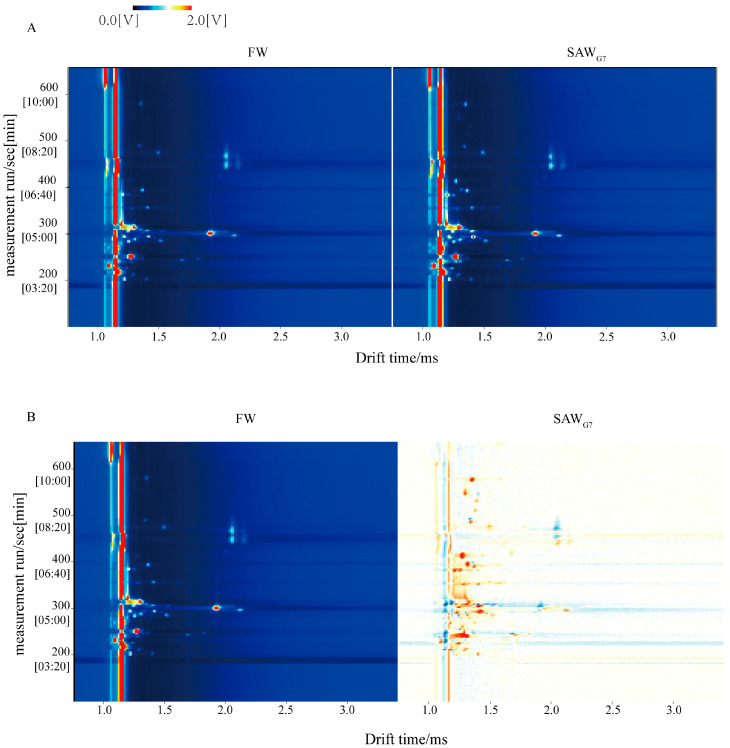
GC-IMS Spectrogram. (**A**) Two-dimensional topographic plots of VOCs in FW and SAW_G7_; (**B**) Comparative difference spectra of VOCs in FW and SAW_G7_, where SAW_G7_ spectra are presented as differential signatures relative to FW. A white background indicated that the compound was consistent with the reference; red and blue backgrounds indicated compounds with higher and lower signal intensities than the reference, respectively. Values are obtained from three biological replicates.

GC-IMS was employed to qualitatively analyze the VOCs in SAW_G7_ and FW to explore their changing patterns further. The two-dimensional (2D) spectrum featured a blue background, with vertical and horizontal axes representing the retention time (Rt) and drift time (Dt) of VOCs during GC separation, respectively [23]. A vertical line at *x* = 1.0 marked the normalized reactive ion peak (RIP), and points to the right of the RIP corresponded to individual VOCs. Peak intensity, calculated based on VOC peak volume, was color-coded: white indicated low intensity, while red denoted high intensity. Figure 2A showed that Rt and Dt values for VOCs in all samples ranged from 200–600 s and 1.00–2.50 ms, respectively. To compare volatile flavor substances between SAW_G7_ and FW, the topographical plot of FW was used as a reference, and SAW_G7_ plots were subtracted from it. In the resulting differential plot, white regions indicated equivalent concentrations in both samples, red dots signified higher concentrations in SAW_G7_ relative to FW, and blue areas represented lower concentrations. Figure 2B revealed a predominance of red dots, highlighting distinct differences in volatile flavor profiles between SAW_G7_ and FW. Additionally, SAW_G7_ exhibited a higher total volatile flavor content than FW.

**Figure 3:**
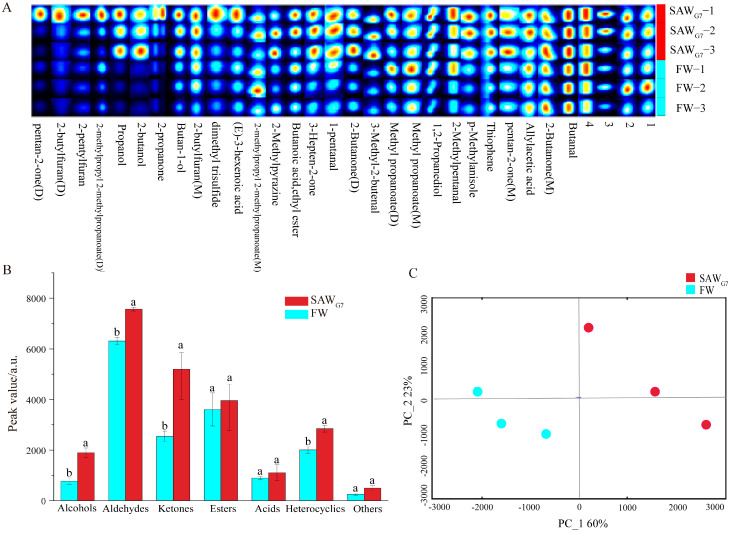
Analysis of VOCs in FW and SAW_G7_ by GC-IMS. (**A**) Fingerprint plot of VOCs from FW and SAW_G7_. Each row corresponds to an individual sample, while each column denotes a specific compound. Designations include Monomer (M) and Dimer (D). (**B**) Comparative quantification of VOCs peak volume between FW and SAW_G7._ Different letters indicate significant differences (*p* < 0.05), whereas identical letters signify no significant differences (*p* > 0.05). Error bars represent standard deviation. (**C**) PCA scores plot based on GC-IMS data for FW and SAW_G7_.

To illustrate differences in VOCs between SAW_G7_ and FW, a VOC fingerprint spectrum was generated using the Gallery Plot plugin (Figure 3A). The fingerprint revealed distinct changes in volatile profiles, with propanol and 2-butanol identified as characteristic flavor compounds in SAW_G7_. Several substances exhibited significant increases following long-term selective breeding for salinity-alkalinity tolerance, including pentan-2-one (M), thiophene, p-methylanisole, 3-methyl-2-butenal, 2-butanone (D), pentanal, 3-hepten-2-one, 2-methylpyrazine, 2-pentylfuran, and 1-butanol.

A total of 32 peaks were detected in SAW_G7_ and FW, corresponding to 32 substances (including monomers and dimers). These included 4 alcohols, 4 aldehydes, 2 acids, 5 esters, 4 heterocyclics, 7 ketones, 2 others (p-Methylanisole and dimethyl trisulfide), and 4 unknowns (Table S2). The qualitative analysis of VOCs in SAW_G7_ and FW is presented in Figure 3B. No significant differences were observed between SAW_G7_ and FW in esters, acids, and others (*p* > 0.05). However, alcohols, aldehydes, ketones, and heterocyclics were significantly elevated in SAW_G7_ compared to FW (*p* < 0.05). Aldehydes represented the highest proportion of VOCs in both samples, followed by ketones and esters, with ketones exhibiting the most pronounced differences between SAW_G7_ and FW. Two organic acids (*(E)*-3-hexenoic acid and allylacetic acid) were identified. PCA was performed to pinpoint molecules with significant inter-sample variation. As shown in Figure 3C, PC1 and PC2 explained 60% and 23% of the variance, respectively, cumulatively accounting for 83% of the total variance, confirming PCA’s efficacy in distinguishing SAW_G7_ and FW. However, GC-IMS is primarily suited for trace VOC detection, and its semi-quantitative results lack absolute quantitation. Thus, complementary GC-MS analysis was conducted to validate and quantify VOC content changes between SAW_G7_ and FW.

### 3.3. VOCs in FW and SAW_G7_ Identified by GC-MS

**Figure 4:**
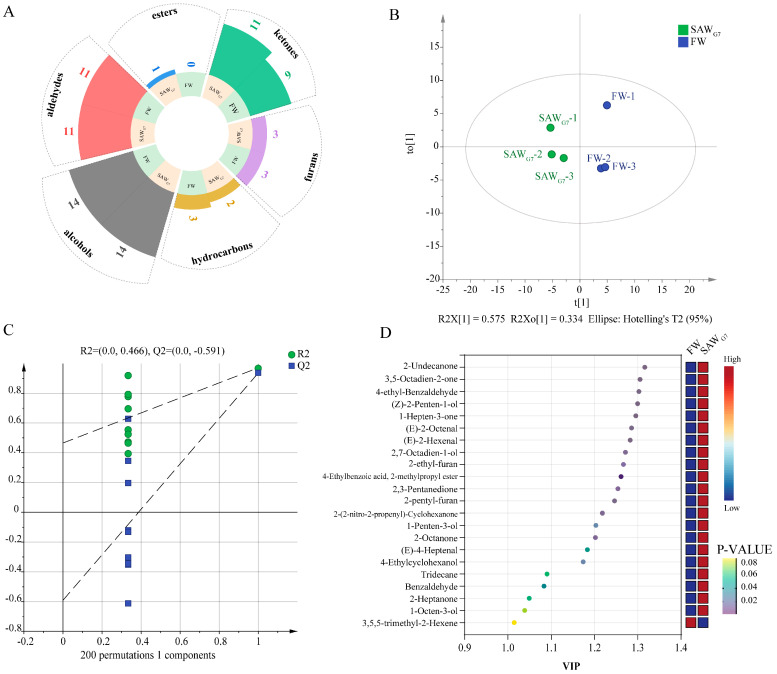
GC-MS analysis. (**A**) The types and quantities of VOCs in FW and SAW_G7_. (**B**) The OPLS-DA score plot of VOCs in FW and SAW_G7_. (**C**) Permutation testing (200) of the OPLS-DA model. (**D**) The key VOCs (VIP > 1.0) identified by OPLS-DA.

A total of 43 VOCs were detected in FW and SAW_G7_ via GC-MS (Table S3). The VOC profiles comprised alcohols (14), aldehydes (11), ketones (11), esters (1), furans (3), and hydrocarbons (3) (Figure 4A). To identify differential VOCs between the two groups, an orthogonal partial least squares discriminant analysis (OPLS-DA) model was established based on GC-MS data [24]. The OPLS-DA score plot (Figure 4B) revealed clear separation of FW and SAW_G7_ within the 95% confidence interval (elliptical region), indicating that long-term saline-alkali adaptation significantly altered tilapia VOC profiles. The model demonstrated strong goodness-of-fit (cumulative *R^2^X* = 0.909, *R^2^Y* = 0.966) and predictive ability (Q^2^ = 0.937). Permutation testing (200 iterations; Figure 4C) confirmed robustness, with a steep regression slope (Q^2^-intercept = −0.591), ruling out overfitting.

Differential VOCs contributing to group separation were screened by integrating variable importance in projection (VIP) scores and *p*-values (threshold: VIP > 1.0, *p* < 0.05; Figure 4D) [25]. Eighteen discriminant compounds were identified, with ketones, ranked by VIP value, contributing most significantly to flavor profile differences. Notably, 2-undecanone and 2-(2-nitro-2-propenyl)-cyclohexanone were exclusively detected in SAW_G7_, while 3,5-octadien-2-one, 1-hepten-3-one, and 2,3-pentanedione concentrations were markedly higher in SAW_G7_ than in FW. These ketones are associated with fresh fruity aromas and sweet, creamy sensations [26]. Among the discriminant VOCs, unsaturated aldehydes predominated, including 4-ethylbenzaldehyde (almond, burnt sugar notes) and (*E*)-2-octenal (fatty, citrus, and green nuances), which contributed to SAW_G7_’s distinct flavor due to their low odor thresholds [27,28]. Unsaturated alcohols, such as (*Z*)-2-penten-1-ol (green, almond), 2,7-octadien-1-ol (fruity, mushroom), and 1-penten-3-ol (vegetal, meaty), also increased significantly in SAW_G7_, likely derived from the degradation of polyunsaturated fatty acids [29,30]. A single ester, 2-methylpropyl 4-ethylbenzoate, was uniquely present in SAW_G7_, enhancing its pleasant odor. Additionally, 2-ethylfuran (caramel sweetness) and 2-pentylfuran (green fruity notes), key furans in aquatic products, further enriched SAW_G7_’s flavor complexity [31].

The origin of muscle VOCs in tilapia is consistent with established lipid, amino acid, and nucleotide catabolism pathways [32,33]. Saline-alkaline acclimation likely intensified enzymatic lipid oxidation processes, as evidenced by increased aldehydes, ketones, and alcohols that were typical enzymatic byproducts. This observation confirms findings by Su et al., who reported altered enzyme activities in tilapia under salt-alkalinity stress [12]. A total of 23 fatty acids were identified in the muscle tissues of FW and SAW_G7_ tilapia groups (Table 1). Among these, palmitic acid (C16:0), a saturated fatty acid, was the most abundant, with its level being significantly higher in FW than in SAWG_7_. For unsaturated fatty acids, linoleic acid (C18:2N6C) and oleic acid (C18:1N9C) were present at relatively high concentrations and were also significantly elevated in FW. Overall, SAW_G7_ exhibited higher saturated fatty acid (SFA) content but lower monounsaturated fatty acid (MUFA) levels compared to FW. In contrast, polyunsaturated fatty acid (PUFA) content showed little variation between the two groups. Notably, the combined (EPA + DHA) content in SAW_G7_ was 2.88 times that of FW, and the n-3/n-6 ratio was 3.05 times higher. Fatty acids serve as major energy reserves in fish, supporting substantial energy consumption during osmoregulation. The marked increase in EPA and DHA in SAW_G7_ may be attributed to enhanced synthesis and retention of these fatty acids under saline-alkaline conditions, or to the preferential catabolism of non-essential fatty acids, leading to relative enrichment of EPA and DHA. These findings highlight substantial flavor divergence between SAW_G7_ and FW, underscoring the pivotal role of saline-alkaline aquaculture environments in shaping tilapia flavor profiles.

### 3.4. Identification of Key Aroma Compounds in Two Species of Tilapia by OAV

The role of individual VOCs in shaping aroma profiles was evaluated using the odor activity value (OAV), a parameter that incorporates both the concentration of each compound and its corresponding odor threshold. Compounds exhibiting OAVs greater than 1 were identified as key contributors to aroma, based on their potential for higher perceptual intensity. The OAVs for these significant odorants, along with their odor descriptors and thresholds, are summarized in Table 2.

Based on OAV evaluation, a total of nine compounds with significant contributions (OAV > 1) were identified, including two alcohols (1-Octen-3-ol, 1-Heptanol), five aldehydes (Pentanal, Hexanal, Heptanal, Octanal, Nonanal), and two ketones (1-Hepten-3-one, 2,5-Octanedione). SAW_G7_ samples exhibited markedly higher OAVs for key compounds such as 1-octen-3-ol and hexanal, indicating a stronger mushroom and grassy-green odor. This elevation is likely linked to increased oxidative degradation of lipids under saline-alkaline stress. In contrast, FW tilapia showed a slightly higher OAV for nonanal, contributing to a relatively enhanced fatty and citrus note, which may relate to different lipid composition or oxidation pathways in freshwater conditions. Additionally, heptanal and octanal were more prominent in SAW_G7_, further reinforcing its greener and sharper aroma profile. These compositional shifts suggest that saline-alkaline adaptation intensifies green and mushroom notes, whereas freshwater cultivation favors a milder, slightly fatty aroma, directly shaping the distinct flavor impressions of tilapia from the two habitats.

### 3.5. Comparative Analysis of E-Nose, GC-IMS, and GC-MS Results

Our multi-platform analytical approach consistently revealed distinct volatile profiles between FW and SAW_G7_. The differential response of the electronic nose, particularly in sensors selective for alcohols, ketones, and aldehydes, is directly attributable to the significantly elevated concentrations of these compounds in SAW_G7_, as quantified by both GC-IMS and GC-MS.

Regarding compound detection overlap, several key odor-active molecules were consistently identified across multiple platforms. Hexanal and 1-octen-3-ol were simultaneously detected by GC-IMS and GC-MS, with their elevated concentrations in SAW_G7_ samples corroborated by significantly enhanced responses in the corresponding E-nose sensors. These compounds, characterized by moderate molecular weights and adequate volatility, demonstrated ideal analytical properties for both separation-based detection systems. Their significant OAVs further validated their crucial roles in flavor differentiation.

The technological divergence in compound detection primarily stemmed from inherent methodological characteristics. GC-IMS exclusively identified trace-level short-chain alcohols and heterocyclics such as 2-methylpyrazine and propanol, attributable to its superior sensitivity toward polar molecules and capacity to resolve compound dimers. Conversely, GC-MS uniquely detected larger molecules, including 2-undecanone and 4-ethylbenzaldehyde, benefiting from its higher chromatographic resolution and robust mass spectral identification capabilities. The E-nose, while non-specific in compound identification, provided crucial real-time validation of sensory-relevant VOC mixtures through its cross-reactive sensor patterns.

This multi-platform integration achieved comprehensive flavor profile characterization: GC-IMS captured the rapid dynamics of trace polar compounds, GC-MS delivered definitive identification and quantification of key odorants, whereas the E-nose bridged the gap between instrumental data and sensory perception. The consistent confirmation of aldehyde and alcohol enrichment across all three platforms, coupled with their significant OAVs, provides compelling evidence for lipid oxidation-derived flavor formation in saline-alkaline-adapted tilapia, thereby establishing a robust analytical foundation for understanding environmental impacts on aquatic product quality.

## 4. Conclusions

Through integrated analytical approaches, this study deciphered the flavor differentiation between SAW_G7_ and FW. Multi-platform analyses (E-nose, GC-IMS, and HS-SPME-GC-MS) collectively demonstrated that SAWG_7_ exhibited significantly enriched VOCs, particularly aldehydes, ketones, alcohols, and furans (e.g., 4-ethylbenzaldehyde, 2-undecanone, 2,7-Octadien-1-ol, 2-pentylfuran), which are strongly associated with lipid oxidation under saline-alkaline stress. PCA and OPLS-DA models confirmed distinct odor profiles between groups, with SAWG_7_ displaying heightened flavor complexity attributed to low-threshold, sensorially impactful VOCs. These findings reveal that saline-alkaline adaptive breeding significantly changes tilapia’s flavor profiles, providing a valuable theoretical foundation and practical insights for refining selective breeding strategies in saline-alkaline-tolerant tilapia. Future studies should integrate lipidomics, transcriptomics, and sensory relevance to unravel biosynthetic pathways of key VOCs and clarify how environmental-genetic interactions modulate flavor metabolism, thereby guiding precision breeding for enhanced product appeal.

## Figures and Tables

**Table 1:** Comparison of fatty acid composition in the muscle of FW and SAW_G7._

Fatty Acid	FW (%)	SAW_G7_ (%)
C4:0	15.80 ± 4.44 a	16.81 ± 13.17 a
C14:0	0.96 ± 0.09 a	0.95 ± 0.15 a
C15:0	0.25 ± 0.05 b	0.35 ± 0.06 a
C16:0	22.90 ± 0.19 a	19.70 ± 0.93 b
C16:1	2.00 ± 0.26 a	1.90 ± 0.14 a
C17:0	0.30 ± 0.05 b	0.50 ± 0.05 a
C18:0	7.60 ± 0.15 b	8.5 ± 0.28 a
C18:1N9T	0.20 ± 0.03	/
C18:1N9C	21.40 ± 0.36 a	13.90 ± 2.62 b
C19:0	8.00 ± 0.16 b	15.70 ± 0.15 a
C18:2N6C	21.50 ± 0.20 a	16.50 ± 0.63 b
C20:0	0.20 ± 0.04 b	0.30 ± 0.03 a
C18:3N6	0.90 ± 0.01 a	0.30 ± 0.08 b
C20:1	1.10 ± 0.22 a	1.00 ± 0.05 a
C18:3N3	1.20 ± 0.10 a	1.00 ± 0.06 b
C20:2	1.60 ± 0.05 a	1.10 ± 0.02 b
C22:0	0.30 ± 0.01 a	0.30 ± 0.05 a
C20:3N6	1.70 ± 0.04 a	0.80 ± 0.04 b
C20:3N3	0.30 ± 0.03 a	0.30 ± 0.04 a
C20:4N6	5.00 ± 0.25 a	4.80 ± 0.03 a
C20:5	0.20 ± 0.02 b	0.70 ± 0.17 a
C24:1	/	1.50 ± 0.13
C22:6NS	3.90 ± 0.11 b	11.10 ± 0.30 a
SFA	48.00 ± 0.58	53.48 ± 1.65
MUFA	21.06 ± 0.17	15.51 ± 0.59
PUFA	30.94 ± 0.09	31.01 ± 0.15
EPA + DHA	4.10	11.80
n-3/n-6(%)	0.19	0.58

FW and SAW_G7_: Different letters indicate significant differences (*p* < 0.05), whereas identical letters signify no significant differences (*p* > 0.05).

**Table 2:** Volatile organic compounds with OAV > 1 in tilapia from two culture origins.

	Volatile Compounds	Threshold	OAV	
		(μg/kg)	SAW_G7_	FW
alcohols	1-Octen-3-ol	1.5 [34]	57.88	27.2
	1-Heptanol	3 [35]	2.86	1.57
aldehydes	Pentanal	12 [36]	1.72	1.03
	Hexanal	5 [34]	88.80	51.54
	Heptanal	3 [36]	6.530	3.66
	Octanal	0.7 [37]	20.45	14.41
	Nonanal	1 [36]	22.10	22.77
ketones	1-Hepten-3-one	0.04 [38]	19.25	4.50
	2,5-Octanedione	29 [35]	1.12	0.72

## Data Availability

The original contributions presented in this study are included in the article/Supplementary Material. Further inquiries can be directed to the corresponding authors.

## Supplementary Materials

The following supporting information can be downloaded at: https://www.mdpi.com/article/10.3390/foods14223946/s1, Table S1: The name of the sensor and its response compound; Table S2: List of VOCs Identified by GC-IMS in SAWG7 and FW; Table S3: List of VOCs Identified by GC-MS in SAWG7 and FW.
