# Supplementary material for: Comparative Analysis of Volatile Organic Compounds in Freshwater-Cultured and Saline–Alkaline Selectively Bred Tilapia Using Electronic Nose, GC-IMS, and HS-SPME-GC-MS
Source: Foods. 2025 Nov 18;14(22):3946. doi: 10.3390/foods14223946 (PMC12651432; doi:10.3390/foods14223946)
Supplement: Supplementary file 1 [file foods-14-03946-s001.zip › foods-3952669-supplementary.pdf]

Comparative analysis of volatile organic compounds in freshwater-cultured and saline-alkaline selective breeding Tilapia using electronic nose, GC-IMS, and HS-SPME-GC-MS

Zhi Wang[1#], Yi Yang[1#], Dongxue Zhang[1], Jiashu Li[1], Longsheng Zhang[2], Yan Zhao[2], Jinliang Zhao[2], Junling Zhang[*2], Jikui Wu[*1, 3]

[1] Shanghai Engineering Research Center of Aquatic-Product Processing & Preservation, Shanghai Ocean University, Shanghai 201306, China

[2] Key Laboratory of Exploration and Utilization of Aquatic Genetic Resources, Ministry of Education; Shanghai Collaborative Innovation Center for Aquatic Animal Genetics and Breeding; Shanghai Ocean University, Shanghai, 201306, China

[3] Laboratory of Quality and Safety Risk Assessment for Aquatic Product on Storage and Preservation (Shanghai), Ministry of Agriculture, Shanghai Ocean University, Shanghai 201306, China

*Corresponding author: Tel. +86-021-61900753; +86-021-61900437

Email address: jkwu@shou.edu.cn; jlzhang@shou.edu.cn.

Address: No. 999, Hucheng Ring Road, Pudong New Area, Shanghai, China.

[#] These authors contributed equally

TableS1. The name of the sensor and its response compound

Sensor serial number	Sensor name	Response substance types
1	LY2/LG	Sensitive to chlorine, fluorine, nitrogen oxides, and sulfides
2	LY2/G	Ammonia, amines, and carbonyl compounds
3	LY2/AA	Ethanol, acetone, and ammonia
4	LY2/GH	Ammonia and amine compounds
5	LY2/gCTL	Hydrogen sulfide
6	LY2/gCT	Propane and butane
7	T30/1	Polar compounds and hydrogen chloride
8	P10/1	Non-polar: hydrocarbons, ammonia, and chlorine
9	P10/2	Non-polar: methane and ethane
10	P40/1	Fluorine and chlorine
11	T70/2	Toluene, xylene, and carbon monoxide
12	PA/2	Ethanol, ammonia, and amine compounds
13	P30/1	Hydrocarbons, ammonia, and ethanol
14	P40/2	Chlorine, hydrogen sulfide, and fluorides
15	P30/2	Hydrogen sulfide and ketones
16	T40/2	Chlorine
17	T40/1	Fluorine
18	TA/2	Ethanol

TableS2. List of VOCs Identified by GC-IMS in SAW_{G7} and FW

compounds	CAS#	Formula	MW	RI	RT[sec]	Dt [a. u.]	Peak Volume (a.u.)	
							SAW_{G7}	FW
2-Methylpyrazine	C109080	$C_5H_6N_2$	94.1	1292.6	804.132	1.10063	378.78±89.24	245.29±59.32
2-pentylfuran	C3777693	$C_9H_{14}O$	138.2	1261.6	753.312	1.25779	125.54±71.36	59.06±4.84
Butan-1-ol	C71363	$C_4H_{10}O$	74.1	1157.3	579.038	1.18406	610.29±75.18	321.82±57.79
2-methylpropyl 2-methylpropanoate(M)	C97858	$C_8H_{16}O_2$	144.2	1097.7	474.361	1.30525	863.81±745.21	503.12±153.03
2-butylfuran	C4466244	$C_8H_{12}O$	124.2	1107.7	490.495	1.17493	245.55±9.83	170.39±40.33
2-methylpropyl 2-methylpropanoate(D)	C97858	$C_8H_{16}O_2$	144.2	1094.1	469.442	1.79628	1247.33±256.81	1437.66±434.31
Propanol	C71238	C_3H_8O	60.1	1050.4	413.685	1.11455	681.93±74.93	120.41±10.66
2- butanol	C78922	$C_4H_{10}O$	74.1	1036	396.861	1.15394	225.14±31.79	49.93±1.34
Butanoic acid, ethyl ester	C105544	$C_6H_{12}O_2$	116.2	1034	394.595	1.19882	704.99±159.16	564.18±82.99
(E)-3-hexenoic acid	C1577180	$C_6H_{10}O_2$	114.1	996.7	354.485	1.22822	567.39±306.11	387.2±51.83
pentan-2-one(D)	C107879	$C_5H_{10}O$	86.1	998.8	356.365	1.41494	262.4±313.02	81.74±20.89
3-Hepten-2-one	C1119444	$C_7H_{12}O$	112.2	929.6	306.28	1.22876	894.81±56.01	484.09±74.11
1-pentanal	C110623	$C_5H_{10}O$	86.1	933.2	308.729	1.18552	193.39±28.15	116.31±17.35
2-Butanone(M)	C78933	C_4H_8O	72.1	910.4	293.728	1.06911	893.95±141.39	779.82±74.47
Methyl propanoate(M)	C554121	$C_4H_8O_2$	88.1	894.3	283.636	1.09566	554.68±16.48	598.24±101.08
Allylacetic acid	C591800	$C_5H_8O_2$	100.1	901.7	288.223	1.14496	536.88±74.75	507.22±23.34
2-Butanone(D)	C78933	C_4H_8O	72.1	912	294.772	1.24227	1304.49±220.96	603.38±51.28
Methyl propionate(D)	C554121	$C_4H_8O_2$	88.1	896.4	284.903	1.32382	584.01±172.93	500.77±157.16
2-n-Butylfuran	C4466244	$C_8H_{12}O$	124.2	887.4	279.377	1.16983	111.97±75.32	55.67±12.13
Butanal	C123728	C_4H_8O	72.1	838.4	251.133	1.10904	6734.78±56.45	5786.84±185.85
2-propanone	C67641	C_3H_6O	58.1	820.8	241.635	1.11662	1632.9±1143.57	444.41±105.57
3-Methyl-2-butenal	C107868	C_5H_8O	84.1	789.5	225.719	1.08825	346.88±88.8	185.08±3.75
1,2-Propanediol	C57556	$C_3H_8O_2$	76.1	759.7	211.516	1.12743	378.5±50.55	277.49±71.08
2-Methylpentanal	C123159	$C_6H_{12}O$	100.2	738.2	201.864	1.22327	297.89±55.3	225.64±75.61
p-Methylanisole	C104938	$C_8H_{10}O$	122.2	1024.7	384.041	1.09666	355.72±29.01	172.78±26.48
Thiophene	C110021	C_4H_4S	84.1	1025.4	384.826	1.05076	1983.95±75.48	1477.21±97.35
pentan-2-one(M)	C107879	$C_5H_{10}O$	86.1	997	354.743	1.1241	208.55±15.5	148.51±1.28
dimethyl trisulfide	C3658808	$C_2H_6S_3$	126.3	998	355.552	1.30656	144.4±85.7	82.82±17.34
1	unidentified	*	0	741.2	203.191	1.06798	1538.97±190.56	1489.69±428.69
2	unidentified	*	0	1098.1	474.974	1.55917	676.77±77.47	609.57±138.09
3	unidentified	*	0	1080	450.633	1.79571	787.4±214.73	474.56±97.93
4	unidentified	*	0	916.2	297.465	1.85428	1037.92±75.62	920.38±124.25

TableS3. List of VOCs Identified by GC-MS in SAW_{G7} and FW

	Metabolite	formula	RT(min)	m/z
alcohols	1-Penten-3-ol	$C_5H_{10}O$	20.162	86.073
	1-Pentanol	$C_5H_{12}O$	23.226	88.089
	1-Hexanol	$C_6H_{14}O$	26.432	102.104
	1-Octen-3-ol	$C_8H_{16}O$	29.176	128.12
	1-Heptanol	$C_7H_{16}O$	29.346	116.12
	1-Hexanol, 2-ethyl-	$C_8H_{18}O$	30.285	130.136
	Cyclohexanol, 2,4-dimethyl-	$C_8H_{16}O$	31.706	128
	1-Octanol	$C_8H_{18}O$	32.047	130.136
	Z-2-Dodecenol	$C_{12}H_{24}O$	33.469	184
	3-Ethyl-4-nonanol	$C_{11}H_{24}O$	34.535	172
	2,7-Octadien-1-ol	$C_8H_{14}O$	35.225	126
	4-Ethylcyclohexanol	$C_8H_{16}O$	29.744	128
	2-Penten-1-ol, (Z)-	$C_5H_{10}O$	25.422	86.073
	1,5-Pentanediol, 3-methyl-	$C_6H_{14}O_2$	34.415	118.099
aldehydes	Pentanal	$C_5H_{10}O$	11.489	86.073
	Hexanal	$C_6H_{12}O$	16.43	100.089
	Heptanal	$C_7H_{14}O$	20.581	114.104
	Octanal	$C_8H_{16}O$	24.363	128.12
	Nonanal	$C_9H_{18}O$	27.669	142.136
	5-Ethylcyclopent-1-enecarboxaldehyde	$C_8H_{12}O_2$	28.529	124
	2-Octenal, (E)-	$C_8H_{14}O$	28.813	126.104
	Benzaldehyde	C_7H_6O	31.621	106.042
	Benzaldehyde, 4-ethyl-	$C_9H_{10}O$	36.206	134.073

	Metabolite	formula	RT(min)	m/z
	4-Heptenal, (E)-	$C_7H_{12}O$	22.927	112.089
	2-Hexenal, (E)-	$C_6H_{10}O$	22.06	98.073
ketones	2,3-Pentanedione	$C_5H_8O_2$	15.605	100.052
	2-Heptanone, 6-methyl-	$C_8H_{16}O$	22.593	128.12
	3-Octanone	$C_8H_{16}O$	23.155	128.12
	1-Hepten-3-one	$C_7H_{12}O$	24.839	112
	2,5-Octanedione	$C_8H_{14}O_2$	25.557	142.099
	6,7-Dodecanedione	$C_{12}H_{22}O_2$	34.294	198.162
	2-Octanone	$C_8H_{16}O$	24.334	128.12
	3,5-Octadien-2-one	$C_8H_{12}O$	31.308	124.089
	2-Heptanone	$C_7H_{14}O$	22.593	128.12
	2-(2-nitro-2-propenyl)- Cyclohexanone	$C_{18}H_{19}NO_3$	30.967	183
	2-Undecanone	$C_{11}H_{22}O$	33.228	170.167
esters	4-Ethylbenzoic acid, 2-methylpropyl ester	$C_{21}H_{28}O2$	38.623	206.131
furans	2-(1-Hydroxycyclohexyl)-furan	$C_{10}H_{14}O_2$	39.696	166
	Furan, 2-pentyl-	$C_9H_{14}O$	22.166	138.104
	Furan, 2-ethyl-	C_6H_8O	10.252	96.058
hydrocarbons	Tridecane	$C_{13}H_{28}$	24.192	184.219
	2-Hexene, 3,5,5-trimethyl-	C_9H_{18}	30.192	126.141
	4-Cyanocyclohexene	C_7H_9N	32.701	107.073
